# Pinpointing Cell Identity in Time and Space

**DOI:** 10.3389/fmolb.2020.00209

**Published:** 2020-08-14

**Authors:** Anca F. Savulescu, Caron Jacobs, Yutaka Negishi, Laurianne Davignon, Musa M. Mhlanga

**Affiliations:** ^1^Division of Chemical, Systems & Synthetic Biology, Faculty of Health Sciences, Institute of Infectious Disease & Molecular Medicine, University of Cape Town, Cape Town, South Africa; ^2^SAMRC/NHLS/UCT Molecular Mycobacteriology Research Unit, Department of Pathology, Institute of Infectious Disease and Molecular Medicine, University of Cape Town, Cape Town, South Africa; ^3^Wellcome Centre for Infectious Diseases Research in Africa, University of Cape Town, Cape Town, South Africa; ^4^Instituto de Medicina Molecular, Faculdade de Medicina da Universidade de Lisboa, Lisbon, Portugal

**Keywords:** spatiotemporal localization, cell subtype classification, spatial transcriptomics, MRNA subcellular localization, cell subtype

## Abstract

Mammalian cells display a broad spectrum of phenotypes, morphologies, and functional niches within biological systems. Our understanding of mechanisms at the individual cellular level, and how cells function in concert to form tissues, organs and systems, has been greatly facilitated by centuries of extensive work to classify and characterize cell types. Classic histological approaches are now complemented with advanced single-cell sequencing and spatial transcriptomics for cell identity studies. Emerging data suggests that additional levels of information should be considered, including the subcellular spatial distribution of molecules such as RNA and protein, when classifying cells. In this Perspective piece we describe the importance of integrating cell transcriptional state with tissue and subcellular spatial and temporal information for thorough characterization of cell type and state. We refer to recent studies making use of single cell RNA-seq and/or image-based cell characterization, which highlight a need for such in-depth characterization of cell populations. We also describe the advances required in experimental, imaging and analytical methods to address these questions. This Perspective concludes by framing this argument in the context of projects such as the Human Cell Atlas, and related fields of cancer research and developmental biology.

## Introduction

Biology inherently requires classification to manage vast amounts of irreducibly complex information. Biological systems are broken up into organs, tissues, cells and molecular pathways, where cells make up the smallest functional units of life. Cells must thus occupy a wide range of phenotypes and morphologies, fulfilling the functional requirements of each of their tissues and niches. Our understanding of this diversity is facilitated by classifying cells across this spectrum as different cell “types” carrying specific molecular signatures. However, cells also exist in dynamic states with some functional plasticity, which presents particular challenges for a reductionist classification approach.

There are two types of classification errors we are at risk of making: either assigning the same identity to two cells when they are different, or conversely, labeling two cells as different when they are functionally identical. To avoid these errors, we need to better understand the parameters that distinguish cells, including the relationship between cell state, function, and identity, to be able to delineate cell sub-types and classes with greater resolution. To illustrate our argument, consider a hypothetical situation: two cells are adjacent to one another in a tissue sample and possess similar levels of the same RNA transcripts. Does this conclude that they are the same cell type? Inversely, if they have differing levels of transcripts, does this mean they are different, or could they simply be in different states or stages in a process? Cells exist in flux, across continuous spectra of differentiation and state. They progress through irreversible processes, such as development or differentiation; oscillatory processes such as the cell cycle and circadian rhythms; as well as reversible transitions between states, including nutritional or disease status. These changes naturally manifest in a cell’s behavior, molecular composition and subcellular organization of various components. It is intuitive to understand processes such as development or differentiation as progressions of cell type or identity. However, it is less clear if cells in distinct but transitory states should be assigned to distinct sub-categories.

As a corollary, by using current techniques that primarily consider cells’ molecular composition (and even their organization within a tissue) we cannot clearly determine where in a process a given cell may be, and thus how similar or distinct one cell identity may be from another.

## Single-Cell Genomics as the Standard Approach to Identify Cell Types and States

**FIGURE 1:**
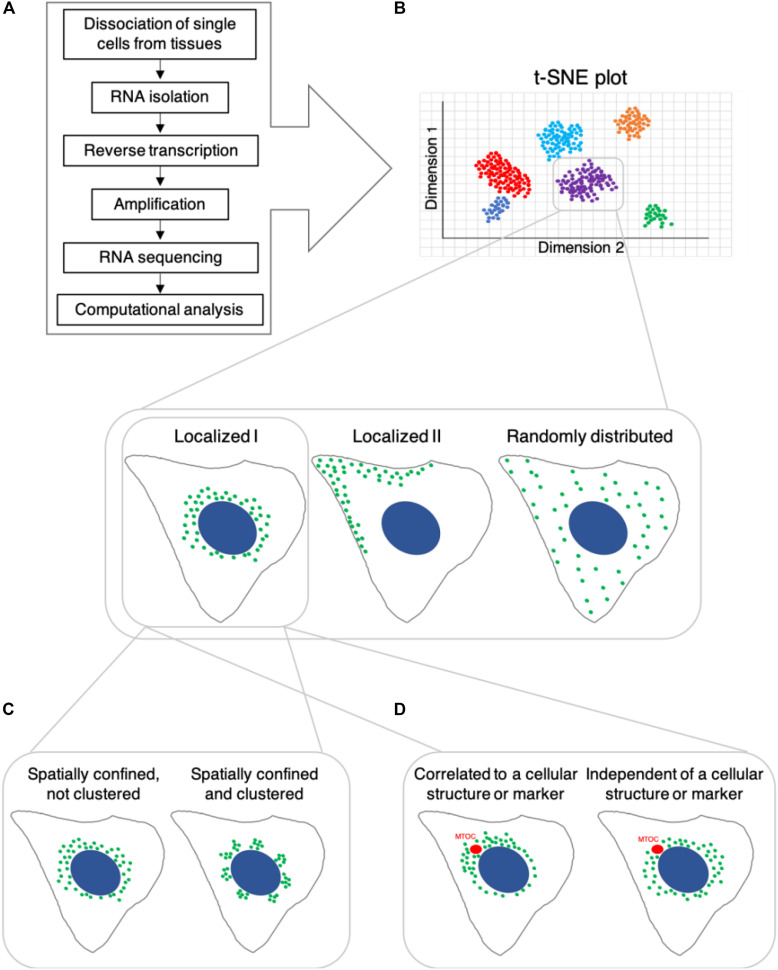
Potential differential spatial distribution and clustering behavior of RNA transcripts in cells, which share the same identity based on single-cell RNA sequencing. **(A)** Single-cell RNA-seq workflow, which typically yields t-SNE plots shown in **(B)**. B Cells that are classified as belonging to the same subtype/group based on RNA transcript count might differ in the subcellular localization of various RNA transcripts. **(C)** Spatially confined RNA transcripts may exhibit non-clustered spatial distribution or localize in clusters. **(D)** Spatial subcellular localization of RNA transcripts may be correlated with or independent of specific cellular structures, organelles or markers.

A cell’s identity is determined by its lineage, present state, and future differentiation or functional potential, as well as its spatial context within a tissue or system (Wagner et al., 2016). Based on the understanding that this identity is reflected in the molecular composition of the cell, single-cell genomics and proteomics have become standard approaches to characterize cell identity and state. Single-cell genomics includes measuring gene expression, typically via RNA sequencing (RNA-seq), as well as epigenetic states and chromatin structure, using approaches which have been adapted to work at the single-cell level (reviewed in Trapnell, 2015; Ludwig and Bintu, 2019; Shema et al., 2019). Single-cell RNA-seq (scRNA-seq) has been rapidly developed and is the most popular approach to identify cell types as it enables classification of cells unbiasedly, based on gene expression patterns and allows to identify novel cell types and subtypes without prior knowledge (for example Darmanis et al., 2015; Grün et al., 2015; Shekhar et al., 2016; Villani et al., 2017; Schroeder et al., 2020). Typical cell-type identification by scRNA-Seq involves dissociating single cells from tissues, followed by isolation of their RNA, reverse transcription, amplification, sequencing and computational analysis (Figure 1A). However, there are several limitations to scRNA-Seq methods: (1) trade-off between the number of cells and data quality; (2) limited measurement of protein expression; (3) noise level and; (4) lack of spatial and temporal information. As technical developments in scRNA-Seq methodology, including computational methods are well documented in other reviews (Hwang et al., 2018; Chen G. et al., 2019; Liao et al., 2020), we discuss representative scRNA-Seq methods and describe their advantages and limitations below.

## Trade-Off Between Number of the Cells and Data Quality

Current scRNA-Seq techniques can be classified based on the single cell capture method: flow cytometry [e.g., Smart-Seq2, (Picelli et al., 2013)], microfluidics [e.g., C1 CAGE (Kouno et al., 2019)], droplets [e.g., 10x Chromium, DroNc-seq (Habib et al., 2017)], Drop-Seq (Macosko et al., 2015), microwell [e.g., Seq Well (Gierahn et al., 2017)], and indexing methods [e.g., sciRNA-Seq (Cao et al., 2017)]. Flow cytometry and microfluidics-based methods enable us to obtain additional biologically relevant data other than gene level expression. For example, Smart-seq detects isoform-level expression and mutations in exon regions, and C1 CAGE quantifies even non-polyadenylated RNA such as enhancer RNAs. Multiomics analysis techniques have been developed for flow cytometry-based methods. For example, scDam&T-seq can analyze RNA level and protein binding sites simultaneously (Rooijers et al., 2019). However, these methods can only measure a maximum amount of a few hundred cells per experiment. As such, they are not suitable for characterization of rare cell populations, as a large number of cells needs to be analyzed on a single cell level in these cases. Droplets, microwell and indexing methods sequence only parts of RNA molecules (in most cases only the 3’ end of the RNA) reversed transcribed by oligo dT. Thus these methods are unable to measure isoform-level expression and non-poly(A)-containing RNAs. However, these methods can measure over 1000 cells per experiment and, as such, are suitable to detect rare cell populations.

## Limited Measurement of Protein Expression

A few recent scRNA-Seq techniques are able to measure protein expression by using oligo conjugated antibodies. For example, CITE-Seq uses oligo-conjugated antibodies against cell surface markers to quantify protein expression levels (Stoeckius et al., 2017). However, currently, CITE-seq allows the measurement of a limited number of proteins on the cell surface. Measuring expression of intracellular proteins by sequencing-based methods remains challenging.

## Noise Level

Compared to bulk RNA-Seq, scRNA-seq data is intrinsically noisy and highly sparse especially due to so-called dropout events - cases in which genes are not detected despite being expressed. Technical variability accounts for approximately 50% of cell-cell variation in expression measurements and affects downstream analyses such as clustering and pseudotime reconstruction. In fact, a large fraction of stochastic allele-specific expression can be explained by technical noise, especially for genes expressed at low and moderate levels (Kim et al., 2015). Although spike-in controls - synthetic nucleic acids used for error calibration that aid in correcting noise, can be used (Lun et al., 2017), these controls are not applicable for droplet-based techniques. Several methods have been developed to denoise data by computationally imputing missing values, including MAGIC, SAVER, scImpute, DeepImpute and others (Huang M. et al., 2018; Li and Li, 2018; van Dijk et al., 2018; Arisdakessian et al., 2019; Luecken and Theis, 2019). Still, it remains challenging to distinguish technical dropout events from biological events. Additionally, it is important to regress out biological events that are not of interest. For example, a novel T cell population was identified only after removal of gene expression governed by the cell cycle (Buettner et al., 2015).

## Lack of Spatial and Temporal Information

Standard scRNA-Seq methods cannot account for spatial or temporal information, with some of the main limitations being the requisite cell-dissociation step, which disrupts the microenvironment and destroys all spatial relationships between cells, and the cell-lysis step, during which spatial information at the subcellular level is lost. Additionally, this latter step induces artifacts and might distort cell type identification (Adam et al., 2017). Recent attempts to address spatial and temporal aspects by RNA-seq have emerged. The first attempt at preserving spatial information in single cell RNA-Seq used *in situ* amplification by padlock probe and RNA sequencing by ligation (Ke et al., 2013). In a method dubbed FISSEQ, Lee et al. (2015) converted RNA in fixed cells and tissues into cross-linked cDNA amplicons, followed by manual sequencing on a confocal microscope. This allowed for enrichment of context-specific transcripts, while preserving tissue and cell architecture. While *in situ* RNA-Seq techniques provide the expression data of highly multiplexed genes with high spatial resolution, analysis of the whole transcriptome remains challenging. On the other hand, non-*in situ* spatial sequencing techniques have been developed. “Spatial transcriptomics” (ST) (Ståhl et al., 2016) and high density spatial transcriptomics (HDST) (Vickovic et al., 2019) make use of a slide printed with an array of reverse transcription oilgo(dT) primers, over which a tissue sample is laid. This allows for imaging, followed by *in situ* untargeted cDNA synthesis and RNA-seq. Read counts can be correlated back to the microarray spot and location within the sample. This has a 2D spatial resolution of ∼100 and 2 μm (or several cells, and less than 1 cell) per spot in ST and HDST, respectively. The ST technique is now commercialized as Visium from 10X genomics. Rodriques et al. (2019) sought to address the question of cell-scale spatial resolution in a tissue by developing SlideSeq. This method functions by transferring RNA from tissue sections onto a surface covered in DNA-barcoded beads with known positions. The positional source of the RNA within the tissue can then be deduced by sequencing. In addition to array-based approaches, a few pioneering methods have been developed to obtain spatial information at cell-cell interactions by computational inference, physical separation by laser microdissection and gentle tissue dissociation (Satija et al., 2015; Moor et al., 2018; Giladi et al., 2020). By combining *in situ* hybridization images, Satija et al. inferred cellular localization computationally. Although this approach is widely applicable, it is challenging to apply to tissues where the spatial pattern is not reproducible, such as in a tumor, or tissues where cells with highly similar expression patterns are spatially scattered across the tissue. While microdissection approaches achieve higher spatial resolution compared to array-based techniques such as Slide-Seq, these approaches only work when the source of spatial variability has a characteristic morphological correlate. Giladi et al. (2020) introduces a new method, PIC-seq, which combines cell sorting of physically interacting cells (PICs) with single-cell RNA sequencing and computational modeling to characterize cell-cell interactions and their impact on gene expression. This approach has a few limitations: doublets might cause mis-identification of cell-cell interaction, and it is not suitable for use on interacting cells that have similar expression profiles. While these non-*in situ* techniques can achieve higher detection sensitivity than *in situ* RNA-Seq at single-cell or nearly single-cell resolution, we suggest that further precise spatial information of RNAs and proteins in the cell is required to fully understand cell state, as exemplified by P granules (see section “Discussion” below).

To understand the transition between cell states and differentiation stages, temporal analyses of the transcriptome and epigenome are essential. The majority of sequencing-based approaches provide only a “snapshot” perspective of any sample, and do not allow us to place the information in the temporal context. To address this limitation, over 70 methods to reconstruct pseudotime have been developed (Reviewed in Saelens et al., 2019; Grün and Grün, 2020), allowing for the characterization of biological processes’ dynamics more accurately than conventional time series of bulk RNA-Seq (Trapnell et al., 2014; Ji and Ji, 2016; Reid and Wernisch, 2016; Qiu et al., 2017; Chen Y. et al., 2019). For example, Monocle (Trapnell et al., 2014), uses single-cell RNA-seq data collected at multiple time points to characterize the temporal aspect of gene expression. This was used to characterize differences in gene expression in differentiation of primary human myoblasts (Trapnell et al., 2014). TSCAN uses RNA-seq data to computationally order cells in a heterogenous population based on the gradual transition of their gene expression (Ji and Ji, 2016). Additionally, SPRING is able to visualize long continuous gene expression topologies, representing a powerful tool to visualize complex differentiation processes such as branching topology of hematopoietic progenitor cells and their differentiation (Weinreb et al., 2018). Another recently developed method, RNA velocity, reconstructs trajectory based on kinetics of nascent and mature mRNA for more solid quantitative foundation (La Manno et al., 2018). These pseudotime reconstruction approaches can aid in unveiling both transitions in gene expression and the dynamics of transcriptional regulation for characterization of gene regulatory networks.

The elucidation of gene regulatory networks can enhance our understanding of complex cellular processes in living cells as a bridge connecting genotypes and phenotypes. Traditional approaches to transcriptome profiling have been successfully used to infer and characterize regulatory networks over time courses such as in differentiation. A notable example is FANTOM5 phase2, which revealed gene regulatory networks by dense time course analyses with CAGE (Arner et al., 2015; Baillie et al., 2017). Network construction from reconstructed pseudotime in scRNA-Seq is challenging due to a combination of biological variation (e.g., stochasticity or bursts) and technical limitations, such as the inability to capture non-poly(A) RNAs. To date, there are only small-scale efforts to derive regulatory networks from single-cell transcriptomics data over time courses. In principle, pseudotime reconstructed from scRNAseq data allows inference of gene-regulatory networks (Aibar et al., 2017). A recent study showed that combining methods for network reconstruction with RNA velocity improves the accuracy of network inference, thus improving the temporal coupling measurement for more accurate reconstruction (Qiu et al., 2020). However, as pseudotime reconstruction methods are based on the assumption that changes in gene expression are continuous or gradual, the approach is not able to capture either drastic or transient changes in transcription that may occur during a process of interest. In addition, we have to keep in mind that scRNA-Seq methods, especially droplet based techniques, might capture doublets, which unless identified and removed, might be mistaken as transient cell states (Kiselev et al., 2019). As with conventional RNA-seq, these approaches are also unable to take into consideration the spatial distribution of RNAs, despite the importance of changes in RNA subcellular distribution during processes such as development and differentiation.

## Spatial and Temporal Information Can Inform a More In-Depth Sub-Classification of Cells

Although there is rapid and ongoing development of sequencing-based technologies, their capabilities to determine high-resolution spatial and temporal information are limited. Emerging evidence indicates that, at a given point in time, not only RNA and protein abundance (Cote et al., 2016), but also differential subcellular distribution of these molecules contributes to a cell’s state and function (for example: Moor et al., 2017). Consider once more our hypothetical situation: can we conclude that two cells, adjacent to one another in a tissue, which possess similar levels of the same RNA transcripts, are the same cell type (Figure 1B). By increasing the spatial resolution at which we assess these samples, we may observe that although at similar concentrations, a particular RNA species could be differentially localized in these cells. For instance, the RNA may be dispersed across the cytoplasm in one cell and locally clustered in the other (Figures 1B,C). The subcellular distribution of mRNA transcripts can determine their binding partners and influence their rate of translation, affecting the cellular concentration and localization of the protein product (Katz et al., 2012, 2016; Moor et al., 2018). This, in turn, can influence the cell’s function and capacity to respond to various environmental cues. Additionally, we may observe spatial positioning of certain transcripts in close association with subcellular landmarks or organelles (Savulescu et al., 2019, reviewed in Suter, 2018; Hughes and Simmonds, 2019 and others) (Figure 1D), including membraneless organelles such as stress granules (for example Khong et al., 2017; Padrón et al., 2019; Wilbertz et al., 2019). This could indicate a functional relationship between the RNA’s cellular role and broader cellular processes, such as cell division, differentiation, polarization etc.’ Further, this may also influence, or be influenced by, cell state or identity.

The significance of RNA/protein subcellular distribution over both space and time can be illustrated using the example of P granules in *C. elegans* development (Brangwynne et al., 2009). Upon polarization of a *C. elegans* single-cell embryo along the anterior-posterior axis, these RNA- and protein-containing condensates shift from a uniform distribution to localize at the posterior half of the cell. This differential distribution determines the germ line and somatic cell fates of the dividing embryo’s daughter cells. Thus, although the function of P granules is not fully understood yet, the spatial distribution of these granules represents a cell-state transition marker (the readiness of the cell to progress to the two-cell stage) as well as a marker for determination of the cell fate (progenitor germ cell and somatic sister cell). Importantly, application of current single-cell sequencing and pseudotime reconstruction methods in this system would not fully reveal the transition of cell state or type, as changes inferred by RNA and protein spatial localization in P granules and possibly additional structures would not be detected. Similar processes involving localization of mRNA transcripts occur in other developmental systems, including determination of spatial patterning in the developing Drosophila embryo (Johnstone and Lasko, 2001) and determination of cell fate in the Xenopus oocyte (King et al., 2005). Such systems highlight a need for technologies capable of accounting for both the temporal and spatial aspects of single-cell genomics for understanding cell types and states.

In a manner similar to mRNA, other species of RNA may be subject to such spatial organization. Our understanding of long non-coding RNA (lncRNA) function and behavior is still in its infancy. However, the intersection of the subcellular organization and function of long non-coding RNAs may contribute to a finer classification of cellular identities and prove to be a particularly interesting field of discovery in the future. Overall, subcellular RNA distribution, as well as interactions between transcripts and cellular structures for trafficking and packaging, may differ between otherwise similar cells. If these differences lead to functional distinctions between the cells, can we still consider them to have the same identity? Expanding on this perspective, potentially hundreds of RNA transcripts in a given experiment may be present at the same level between cells; however these transcripts might be differentially distributed within them. Thus a matrix of thousands of potential combinations of RNA localization patterns may exist, suggesting the possibility of a large array of granularly differentiated cell subtypes that might have previously been classified as belonging to the same group.

While the discussion here has focused on cell classification by transcriptional data, it is important to recognize that proteomic data and the spatial organization of a cell’s protein repertoire could potentially contribute to cell classification decisions. Recent large scale studies indicate that cells that appear genetically identical display various protein levels and subcellular localizations of proteins (Sigal et al., 2006; Breker et al., 2013; Thul et al., 2017; Lu et al., 2018) during differentiation (Balázsi et al., 2011; Rubakhin et al., 2013), in response to environmental stimuli (Narayanaswamy et al., 2009; Balázsi et al., 2011; Breker et al., 2013) or drug treatment (Tkach et al., 2012; Dénervaud et al., 2013; Torres et al., 2016; Itzhak et al., 2017; Shaffer et al., 2017). This could be due to local translation of differentially distributed mRNAs, or post-translational modifications of the protein, inferring differential interactions with binding partners. Similarly to RNA, this phenomena may apply to multiple different protein species. Integrated with the spatial distribution information of RNAs, this could exponentially expand the matrix of cellular organizations, highlighting the potential for in-depth cellular classification for accurately resolving cell states and identities.

The classic approach to study spatial proteomics relies on subcellular fractionation of organelles coupled with mass spectrometry analysis. Additional spatial information at high resolution can be obtained from approaches combining proximity labeling mediated by engineered ascorbic acid peroxidase (APEX) or antibody-mediated affinity purification with mass spectrometry (for example Hein et al., 2015; Huttlin et al., 2017; Lobingier et al., 2017; Paek et al., 2017). This allows for the characterization of the interactome of a protein, based on the assumption that proteins must be in close proximity to be able to interact. As such, it is also indicative of the local spatial proteome. However, these approaches are still in their infancy, and in-depth coverage of the cell proteome has not been completed to date (Reviewed in Lundberg and Borner, 2019). A complementary approach to mass spectrometry-based methods relies on imaging of proteins on a proteome-wide level and at single-cell resolution (Reviewed in Lundberg and Borner, 2019). The Human Protein Atlas (HPA) initiative aims to map the spatial subcellular distribution of all human proteins in all cell types of the human body (Thul et al., 2017; Uhlen et al., 2010; Uhlén et al., 2015; Uhlen et al., 2017). The spatial distribution of an extensive number of proteins has been determined, using antibody labeling, confocal microscopy, and manual and computational image analysis, allowing the detailed classification of subcellular localization of these proteins. Ongoing research is being conducted to further characterize the spatial distribution of proteins, making use of technological advances in multiplexed imaging, endogenous protein tagging, automated fluorescence microscopy and image analysis tools, including deep neural networks (Reviewed in Lundberg and Borner, 2019). It can reasonably be expected that similar large-scale approaches could be applied to the study of RNA subcellular localization, and allow the characterization of the spatial distribution of potentially all cellular RNAs in cell lines and tissues. This would aid in the fine grained subclassification of cell types and states.

## Analytical and Imaging-Based Methods Required to Analyze Spatial Information

Given the complexity of subcellular organization, and a cell’s inherent state of flux, we anticipate that in-depth characterization of the subcellular organization of multiple molecular species across large numbers of cells will require advances in analytical imaging-based methods. Such methods would need (1) the capacity to label multiple RNA transcripts and proteins in a multiplexed manner; (2) acquisition of data at both high spatial resolution and high throughput; and (3) computational frameworks for quantitative image analysis of large, multi-dimensional imaging data sets. The latter would be particularly important to distinguish between the subtle differences in spatial distribution of molecules which could occur between cells. Further, these tools need to be adaptable between cultured cell monolayers, large 3D cultures, including spheroids and organoids, and intact tissue samples. This would ensure the capture of spatial information under controlled conditions and from cells in their native tissue context.

Several labeling and imaging modalities have been developed to meet conditions (1) and (2). Efforts to increase the labeling sensitivity and throughput capacity of hybridization-based techniques have led to the emergence of several sophisticated RNA FISH (fluorescent *in situ* hybridization) techniques, in some cases paired with targeted *in situ* cDNA synthesis and sequencing. Conventional single molecule FISH (smFISH) makes use of multiple short single-stranded DNA oligonucleotide probes, each labeled with a single fluorophore, to target and specifically label mRNA (Raj et al., 2008). A key adaptation for increased RNA FISH labeling capacity has been the use of sequential rounds of multi-color labeling and imaging of the same sample. An intuitive variation of this approach is massively multiplexed cyclic smFISH, such as osmFISH (Codeluppi et al., 2018), which was used to label 33 targeted gene transcripts over 13 rounds of labeling to map the cellular architecture of the mouse neural cortex. Labeling capacity is further boosted by the adoption of FISH probe barcoding approaches, along with sequential labeling, as demonstrated with multiplexed error-robust FISH (MERFISH) (Chen et al., 2015; Moffitt and Zhuang, 2016) and sequential FISH (seqFISH and seqFISH +) (Lubeck et al., 2014; Eng et al., 2019). These techniques begin to approach full-transcriptome imaging, with the capacity to label 100 to 10000 s of RNA species at single cell, subcellular (Lubeck et al., 2014; Chen et al., 2015) or even sub-diffraction (Eng et al., 2019) resolution. STARmap (Wang et al., 2018) uses *in situ* amplification of target-specific probe barcode regions, that can be decoded by 3D sequencing within samples converted to a hydrogel matrix. This allows for the detection of 1000 s of RNA species in large cell numbers in 3D tissue structures. While these approaches can all provide great insight into the functional cellular organization within tissues, they each have varying limitations in the spatiotemporal resolution or throughput available.

Many of these approaches benefit from specialized LabWare and equipment. There is an increasing ease of access to affordable liquid handling systems, driven by open wet-lab solutions such as OpenLH (Gome et al., 2019) and modular Lego-based and 3D-printed injection pumps (for example Almada et al., 2019). Such systems are essential for high-cycle sequential labeling of tens to hundreds of molecular targets, which can be labeled in a single sample without barcoding, as demonstrated with osmFISH (Codeluppi et al., 2018). Automated microscopes allow increased imaging throughput of both sample size and number, however this is typically done at low magnification and resolution. High-resolution visualization of molecular targets is usually limited to the single-cell scale. A new imaging modality termed synthetic aperture optics (i.e., Stellarvision Microscope, Optical Biosystems) (described in Ryu et al., 2006) uses interferometry to increase the effective resolution of low magnification imaging. This drives significantly higher throughput (∼100 s–1000 s of cells) of high resolution (subcellular and up to single-molecule) imaging. Such a system, particularly if coupled with on-line fluid handling for sequential labeling, would be well suited for quantitative spatial characterization of the molecular repertoire of large numbers of individual cells or tissue sections.

Requirement (3), the availability of computational frameworks for quantitative image analysis of large imaging data sets is an area of rapid ongoing development. This spans the full post acquisition pipeline, from basic image processing to spot detection, decoding, quantification, and classification. Quantitative image analysis tools commonly used for image-based phenotypic cell profiling include ways to facilitate feature extraction, data quality control and normalization, dimensionality reduction and clustering from large numbers of cells (described in Caicedo et al., 2017). Many of these techniques could be adapted for the analysis of spatial and temporal characterization by transcriptomics. With the maturation of deep learning technology, there is also extensive potential for the application of artificial neural network-based approaches for image processing and analysis. Deep learning-based approaches are typically best-suited to the processing of large sets of complex data with many parameters, as would be expected of these imaging assays.

Applications of neural network-based approaches include image denoising, segmentation and categorization. Several neural network-based tools are already available for the restoration of images with high noise levels (for example Weigert et al., 2018; Batson and Royer, 2019; Krull et al., 2019). Tools for network-based FISH spot detection have likewise started to emerge (for example Gudla et al., 2017; Mabaso et al., 2018). A common challenge with smFISH applications is the density of signal, particularly in the case of abundant transcripts. This is linked to high levels of background signal, compromising the signal-to-noise ratio (SNR) and our ability to automatically detect and quantify spots. Already-available network-based denoising and spot detection tools could be further adapted for the particularly challenging low SNR and high haze conditions commonly encountered in smFISH. In addition to spot detection, spatially resolved transcriptomics necessitates the ability to distinguish individual and adjacent cells from each other, and a way to characterize the distribution of FISH spots across individual cells. While there are a large number of cell segmentation tools available (reviewed in Meijering, 2012 and Vicar et al., 2019), the automated segmentation of densely packed cells and nuclei, either in a cultured monolayer or intact tissue sections remains a challenge. Potential solutions to this may also lie in machine learning and network-based approaches (for example Al-Kofahi et al., 2018; Schmidt et al., 2018; Berg et al., 2019). Beyond image processing, characterization of cellular FISH spot distribution patterns, including quantification per cellular compartment, could make use of similar approaches to those used for localization pattern classification in spatial proteomics. These include K-nearest neighbor classifiers, support vector machines, artificial neural networks and decision trees (reviewed in Lundberg and Borner, 2019).

As experimental technologies develop and generate high-resolution spatial and temporal cell characterization datasets, ongoing development of tools and platforms to analyze this data will be imperative. Many of the novel image processing and analysis tools described above require optimization for high throughput. In addition, there is a need for development of complete analytical pipelines and frameworks for processing and extracting the complex information and patterns from these imaging data sets. Early iterations of such frameworks can be seen in emerging platforms such as DypFISH (Dynamic patterned FISH) (Savulescu et al., 2019), and Starfish, under development by the SpaceTx consortium in association with the Human Cell Atlas project (described in Perkel, 2019). DypFISH is a recently developed analytical platform for quantitative characterization of the spatial and temporal subcellular distribution of key biomolecules at a single cell level. This system makes use of micropatterning to constrain the architecture of the cell, inferring a reduction in variation of subcellular distribution of mRNA and protein and allowing for high reproducibility. This approach was used to quantify the correlation of mRNA and protein spatial distributions and the MTOC (a key indicator of a cell’s polarity) in mouse fibroblasts, revealing important spatial and temporal differences between mRNA species, as well as within an mRNA species during polarization. This may indicate differential cell state-dependent spatial distribution of important biomolecules. DypFISH may thus be a first step in establishing a more comprehensive approach to the characterization of spatial and temporal information in tissues and other biological systems with high levels of complexity. The Starfish platform seeks to address critical aspects of data handling and pre-processing, as well as spot detection and RNA identification in a flexible manner. This enables the platform to handle data sets from multiple techniques already described here, and extract and compare information across different experiments. More recently, SpatialDB (Fan et al., 2020) has been set up as a manually curated and explorable repository of spatially resolved transcriptomic datasets from multiple techniques. As these analytical platforms develop, we expect that the integration of each of these tools into a single framework, in a modular manner, will be beneficial to researchers seeking to understand cell identity and differences in biology.

Indeed, there is already a parallel drive for the integration of single cell sequencing approaches with imaging-based approaches. RNAscope, which makes use of branched DNA, (Wang et al., 2012, marketed by ACDBio) has been shown to be amenable to multiplexing and image-based transcriptomics, especially when paired with approaches such as automated liquid handling (Battich et al., 2013) and FISH probe barcoding (Xia et al., 2019). More recently, RNAscope has been paired with scRNA seq to demonstrate molecular heterogeneity and cellular dynamics in epidermal wound healing (Haensel et al., 2020). scRNA seq, combined with both ST and targeted *in situ* sequencing, has been used to compile an atlas of the developing human heart (Asp et al., 2019). In another case, ST has been combined with scRNA seq of portions of the same tissue sample, to characterize the tissue architecture in pancreatic ductal adenocarcinoma (Moncada et al., 2020). Computational approaches to allow the integration of these distinct types of data are also rapidly developing. Moncada et al. (2020) made use of multimodal intersection analysis to integrate the image and sequencing data. Other recently advanced analytical methods include the use of probabilistic models (Andersson et al., 2019), supervised learning approaches to mixed-data decomposition (Cable et al., 2020), and SPOTlight (Elosua et al., 2020), which uses non-negative matrix factorization regression models for the deconvolution of ST spot data.

## Discussion

The intricate relationship between a cell’s subcellular molecular organization, its spatiotemporal context within a tissue and system, and its identity and function has a significant impact on our understanding of cell biology. The benefit of high-resolution spatiotemporal cell type characterization, taking into account tissue- to subcellular- scale information, is evident for a number of research fields, including developmental biology (Asp et al., 2019), cancer research (for example Baccin et al., 2020; Moncada et al., 2020 and Yoousuf et al., 2020), and precision medicine (Petitprez et al., 2018). Spatiotemporal characterization of the tumor microenvironment, for example, can provide insight into the composition, organization and functionality of tumor-associated cells, and their roles in tumor development and severity of disease (reviewed in Petitprez et al., 2018). This kind of research also holds the potential for more sophisticated approaches to treating malignant tumors, for example, where cells may have previously underappreciated transitional states that can be targeted. It may also be interesting to assess if differential spatial and temporal distribution of key disease biomarkers, in addition to their expression levels, could be linked to variation in response to treatment between patients. Recent studies at single-cell resolution indicate that subcellular spatiotemporal transcriptomic characterization could also help us understand the molecular basis and progression of certain genetic disorders, such as Arrhythmogenic cardiomyopathy (Boogerd et al., 2019) and cognitive diseases such as Alzheimer’s Disease (Chen W.-T. et al., 2019) and Parkinson’s Disease (Aguila et al., 2019).

The role of cell state is also increasingly appreciated in infection and immunity (reviewed in Kunz et al., 2018), particularly in infectious diseases where immune regulation is key to disease outcomes. For example, the lineage and metabolic state of macrophages can have profound effects in *Mycobacterial tuberculosis* infection (Huang L. et al., 2018, reviewed in Shi et al., 2019). Full characterization of macrophage cell types and states may improve our understanding of, and ability to better treat, TB disease. Transcription-based cell classification is inherent to initiatives such as the Human Cell Atlas project. Here, too, the incorporation of high-resolution spatiotemporal information holds important potential for further biological insights and may greatly enhance the translational benefits of these initiatives. Current cell classification processes will need to be adapted to include this higher granularity of information, and these large-scale projects can be expected to drive the integration of novel experimental and imaging technologies for spatiotemporally resolved characterization. This will include new and advanced analytical approaches and data representation methods. Such developments can highlight and make accessible the wealth of information available by these approaches.

Throughout this Perspective we have emphasized the need to take into account spatiotemporal information when characterizing cell state and identity. As we have discussed, an increasing body of data supports the effect of variation in mRNA/protein expression and subcellular localization in directing cell state and identity. Nevertheless, it is important to acknowledge that not all such variation is necessarily associated with functional changes in cell state. Transcriptional regulation, at a single-cell level in mammalian cells, is probabilistic and intermittent. This leads to production of mRNA transcripts in pulses and can contribute to cell-to-cell heterogeneity (Femino et al., 1998; Costelloe et al., 1999, reviewed in Hume, 2000). This, again, points to the question of how we delineate cell state and identity, using integrated single-cell transcriptomics and tissue-level and subcellular spatial organization data. Multi-scale spatiotemporal information, over a large number of cells and samples, is needed for us to quantitatively assess the extent of variation across tissues and within cells, and to detect rare events and cell types. Assessment of such data should take place in the context of our increasing understanding of basic intracellular processes, and the functions of tissues and disease states being studied. These studies and analyses will be key in fueling important discussions within the field. Pertinently, the different scales of resolution that may apply to different questions should be carefully considered. Single-cell transcriptomics and subcellular spatiotemporal organization contribute to cell state and identity, and can contribute to tissue function. However, this subcellular resolution may not always be necessary to understand the role of individual cells and how they interact with neighboring cells in the context of their tissue. A related discussion is necessary around what “threshold” (or multiple situation-dependent thresholds) of the extent of spatiotemporal variation (detection of which is resolution-dependent) may be considered to delineate functionally distinct states or identities to individual cells.

Accounting for the dynamic states and functional plasticity available to cells has already emerged as important to the classification and characterization of cell types. This will become more widely acknowledged with the parallel development of powerful tools and technology to produce, process and mine the emerging information. These and other necessary developments described here will allow accurate, high-resolution cell classification and improved understanding of the function of different cells in tissues. Taken together, these advancements will provide powerful tools for advances in fundamental biology, biomedical research and related fields.

## Author Contributions

AS, CJ, LD, and MM conceived the idea. AS, CJ, LD, and YN wrote and proofread the manuscript. All authors read and approved the manuscript.

## Conflict of Interest

The authors declare that the research was conducted in the absence of any commercial or financial relationships that could be construed as a potential conflict of interest.
